# Editorial: Legacies of epigenetic perturbations

**DOI:** 10.3389/fcell.2023.1228115

**Published:** 2023-06-13

**Authors:** Simão Teixeira da Rocha, Shau-Ping Lin, Neil A. Youngson

**Affiliations:** ^1^ iBB–Institute for Bioengineering and Biosciences and Department of Bioengineering, Instituto Superior Técnico, Universidade de Lisboa, Lisbon, Portugal; ^2^ Associate Laboratory i4HB Institute for Health and Bioeconomy, Instituto Superior Técnico, Universidade de Lisboa, Lisbon, Portugal; ^3^ Institute of Biotechnology, National Taiwan University, Taipei, Taiwan; ^4^ Bachelor Program of Biotechnology and Food Nutrition, National Taiwan University, Taipei, Taiwan; ^5^ Center for Developmental Biology and Regenerative Medicine, National Taiwan University, Taipei, Taiwan; ^6^ Agricultural Biotechnology Research Center, Academia Sinica, Taipei, Taiwan; ^7^ School of Biomedical Sciences, UNSW Sydney, Sydney, NSW, Australia; ^8^ Centre for Reproductive Health, Department of Molecular and Translational Science, Hudson Institute of Medical Research, Monash University, Clayton, VIC, Australia

**Keywords:** epigenetics, DNA methylation, noncoding RNA, environmental factors, intergenerational inheritance

**FIGURE 1:**
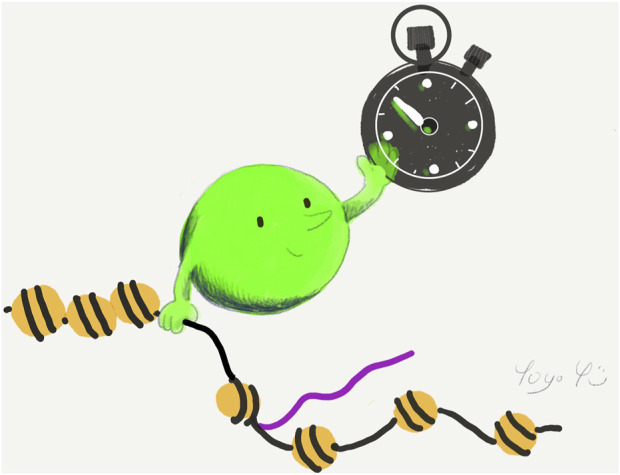
Legacies of epigenetic perturbations. Artwork from Dr. Yoyo Chih-Yun Yu illustrating the legacy that epigenetic modifications may endure in response to developmental/environmental cues.

Transient exposure to specific external cues can have long-lasting effects that persist for decades or even generations. Epigenetic modifications, such as DNA methylation, histone modifications and noncoding RNAs are believed to facilitate these enduring effects (Figure 1). As well as accidental exposures which result in epigenetic change, direct epigenetic interventions through epidrugs, manipulation of the epigenetic apparatus, or cutting-edge locus-specific epigenetic editing tools can leave a legacy which impacts biological processes. Discoveries in this area are being made in cultured cells as well as whole organisms and even populations. As of today, many challenges persist in the field of epigenetic inheritance: Which environmental exposures affect the epigenome and have long-lasting effects? How stable or reprogrammable can an epigenetic mark be? Is there a causal relationship between a particular epigenetic modification and its putative functional consequence? How to disentangle epigenetic from genetic inheritance mechanisms in genetically variable human populations? What are the species-specific differences in mechanisms of transgenerational epigenetic inheritance? This Research Topic devoted to legacies of epigenetic perturbations gives a glimpse on ongoing studies addressing some of these important and challenging questions.

Environmental toxicants are a major area of epigenetic inheritance research, with some of the first examples of environmentally-induced effects on future generations in mammals being shown through exposure of rats to the agricultural fungicide vinclozolin (Anway and Skinner, 2008). Two articles in this Research Topic shed new light in this area. Hartman et al., describe the effects of *in utero* exposure to an insecticide in their study entitled *Epigenetic effects promoted by neonicotinoid thiacloprid exposure*. Whereas Zeng et al., describe the intergenerational effects of adult male mouse exposure to a heavy metal in their study entitled *Differential expression profiles and potential intergenerational functions of tRNA-derived small RNAs in mice after cadmium exposure*.

We wish to highlight two major concepts of legacies of epigenetic perturbations which the Neonicotinoid exposure study illustrates (Hartman et al.). Firstly, the doses administered were 4 times lower than the no-observed-adverse-effect level (NOAEL) dose for females, however still had striking effects on spermatogenesis in male offspring. This is due to the unique environmentally-sensitive gestational epigenetic reprogramming window in developing germ cells. This study therefore emphasises the potential health and fertility risks imposed by current drug safety assessment and classification regulations which may not adequately account for intergenerational effects. Secondly, the study provided mechanistic information on legacies of epigenetic perturbations in the identification of reduced histone modifications and chromosomal-level DNA damage in the adult offspring testes. These two processes provide molecular explanations for developmental and health abnormalities in offspring but also, through DNA mutations, provide an example of permanent genetic aberrations which can perpetuate and accumulate in mammalian populations *ad infinitum*.

The study by Zeng et al. also brings exciting new insight into the mechanisms of intergenerational inheritance, but through tRNA-derived small RNAs (tsRNA) in sperm. Other studies have shown that sperm tsRNAs are environmentally-sensitive epigenetic information carriers, and can induce pathology-associated or even potentially adaptive phenotypes in offspring (Yan and Zhai, 2016). Zeng et al. expanded on the variety of detrimental effects of cadmium exposure on male fertility by identifying 14 exposure-specific sperm tsRNAs. They then undertook experiments that support the hypothesis that these 14 tsRNAs modulate ion transmembrane transport in offspring testes and liver, particularly in organelles (mitochondria and lysosomes). This work raises the possibility of having to treat future populations for ancestral cadmium pollution as well as the exposed generation.

Besides small RNAs, long noncoding RNAs (lncRNAs) are important regulators of epigenetic modifications. They are a heterogeneous class of untranslated RNA molecules with a length over 200 bp. Some of them have relevant roles in biological processes such as gene regulation and chromatin modification during development and cancer. Li et al. review the role of lncRNAs in mitophagy, the process of selective degradation of dysfunctional mitochondria by autophagy, in cancer. Although the role of mitophagy in cancer remains enigmatic, recent studies highlight how cancer cells subvert the mitophagy pathway to their advantage to satisfy their metabolic needs triggered by increased proliferation and invasiveness. In this review, the authors frame their perspective on how lncRNAs mediate the mitochondria-nuclear crosstalk and their implications for anti-cancer therapies. Notably, they discuss the involvement of mitochondria-associated lncRNAs, such as *MALAT1*, in regulating cancer metabolic resetting and the stemness properties of cancer stem cells through mitophagy control. They put forward the idea that lncRNA-based therapy to target mitophagy in cancer cells could leave an epigenetic legacy to treat malignancies, but the road to a complete understanding of the role of lncRNAs in mitophagy is still long.

DNA methyltransferase 3-like (DNMT3L) has been characterized as a critical epigenetic co-factor highly expressed in prospermatogonia, developing oocytes and fetal bovine serum supplemented pluripotent stem cells in culture, that is necessary for proper germ cell function (Liao et al., 2012). The observed meiosis defects in *Dnmt3l* ^−/−^ mice after birth have been predominantly ascribed to the preceding expression of DNMT3L protein starting from the fetal prospermatogonia stage (Zamudio et al., 2015) or, to a lesser degree, from a dormant subpopulation of DNMT3L expression in spermatogonia progenitor cells (Liao et al., 2014; Tseng et al., 2015). These events occur well before the differentiation stage associated with the observed defects in homologous chromosome alignment, thus exemplifying a typical legacy scenario. Apart from germ line studies, our co-editor Prof. Shau-Ping Lin’s team demonstrated that transient ectopic DNMT3L expression for a week in fibroblasts also reinforced chromatin surveillance and halt senescence progression for dozens of passages (over 6 months and still going) (Yu et al., 2020). In this Research Topic, her group’s independently reviewed article demonstrates the importance of these epigenetic inheritance processes for an area of increasing medical significance, cellular therapies. Yang et al., showed that the ability of *Dnmt3l* KO mice derived multipotent mesenchymal stem cells to differentiate *in vitro* into osteocytes was impaired compared to that from wild type littermate, even when the DNMT3L protein itself is not expressed in these cell types (only a potential novel RNA isoform covering the 3′end of the gene can be detected). A broad comparison of transcriptomes, and epigenomic datasets pointed to either persistence of abnormal epigenetic states at a handful of influential developmental regulator genes, or widespread dysregulation of transposable elements as the inheritance mechanism from the DNMT3L expressing pluripotent cells. However, their *in vivo* investigation in *Dnmt3l* KO mice surprisingly revealed beneficial effects on bone in aged female mice, and in male mice exposed to a high-stress lifestyle paradigm. This work highlights the potency, yet complexity and unpredictability of legacies of epigenetic perturbations.

We would like to thank all the submitting authors and the reviewers for this Research Topic. We encourage readers to explore these articles further as they convey many of the major themes and research challenges in the Research Topic of legacies of epigenetic perturbations.
